# Diabetes Management in Patients With Dementia: A Quality Improvement Project to Enhance Staff Confidence, Understanding, and Patient Care

**DOI:** 10.1192/bjo.2025.10400

**Published:** 2025-06-20

**Authors:** Vipassana Bajracharya, Hugh Kelly, Vatsala Mishra, Latha John, Stacey Blackford

**Affiliations:** 1 GKT School of Medicine, King’s College London, London, United Kingdom; 2 Princess Alexandra Hospital, Brisbane, Australia; 3 Oxleas NHS Foundation Trust, London, United Kingdom

## Abstract

**Aims:** This quality improvement project (QIP) aimed to improve nursing staff confidence and understanding of diabetes/management by 40% by the end of February 2024, taking place on a dementia intensive care ward in South East London. This project was led by a medical student and FY2 doctor who noticed significant anxiety and uncertainty amongst healthcare professionals (HCPs) managing a patient’s severe type-2 diabetes, leading to disruption in care as HCPS sought repeated consultation with medics for reassurance prior to implementation of plans, creating an environment of dependency and reduced confidence making independent decisions. Barriers to delivering high-quality care for diabetes in psychiatric services are well-documented and associated with limited understanding of the condition and low confidence in management. Suboptimal management increases risk of diabetic emergencies such as diabetic ketoacidosis/Hyperosmolar Hyperglycaemic State (DKA/HHS), and long-term complications.

**Methods:** Three PDSA cycles were carried out:

PDSA 1: a poster displaying an individualised care plan and insulin regime, alongside an information sheet on diabetes care.

PDSA 2: a teaching session was delivered to nursing staff regarding diabetes care and recognising/managing diabetic emergencies including DKA/HHS.

PDSA 3: further teaching session incorporating feedback from PDSA 2 e.g. using worked examples for staff to apply learning.

Confidence and understanding were assessed using self-reported scales before and after intervention, alongside feedback for potential improvements to subsequent cycles.

**Results:** PDSA 1: Post-intervention feedback showed 47% increase in mean staff confidence in managing diabetes, and 45% increase in understanding of insulin management, exceeding the predictions for PDSA 1.

PDSA 2: Confidence levels improved by 58% and understanding of diabetes management increased by 57%.

PDSA 3: Post-intervention feedback showed mean staff scores for understanding of diabetes increased by 63%. Understanding around blood sugar checks improved an average 38% and most notably, the average score for understanding around ketone checks improved 108% following the teaching session. Confidence levels improved by 91%, and there was an 83% increase in recognition of diabetic emergencies following this presentation.

**Conclusion:** This QIP exceeded its aims and supports the effectiveness of posters and educational sessions in elevating confidence and understanding of diabetes and its management, for the sake of patient care. Worked examples to apply and consolidate new knowledge were the most effective intervention, and the benefits of regular teaching through presentations alongside posters as visual aids are evident. Next steps include consideration around maintaining the change and expanding at directorate/Trust level, and potential rollout for other physical health conditions.

